# Reliability in the Identification of Midbrain Dopamine Neurons

**DOI:** 10.1371/journal.pone.0015222

**Published:** 2010-12-09

**Authors:** Elyssa B. Margolis, Allison R. Coker, Joseph R. Driscoll, Anne-Iris Lemaître, Howard L. Fields

**Affiliations:** 1 Ernest Gallo Clinic and Research Center, University of California San Francisco, Emeryville, California, United States of America; 2 Department of Neurology, University of California San Francisco, San Francisco, California, United States of America; 3 Wheeler Center for the Neurobiology of Addiction, University of California San Francisco, San Francisco, California, United States of America; VU University, Netherlands

## Abstract

Brain regions typically contain intermixed subpopulations of neurons with different connectivity and neurotransmitters. This complicates identification of neuronal phenotypes in electrophysiological experiments without using direct detection of unique molecular markers. A prime example of this difficulty is the identification of dopamine (DA) neurons in the midbrain ventral tegmental area (VTA). Although immunocytochemistry (ICC) against tyrosine hydroxylase (TH) is widely used to identify DA neurons, a high false negative rate for TH ICC following *ex vivo* electrophysiology experiments was recently reported, calling into question the validity of comparing DA and non-DA VTA neurons based on post-hoc ICC. However, in whole cell recordings from randomly selected rat VTA neurons we have found that TH labeling is consistently detected in ∼55% of neurons even after long recording durations (range: 2.5–150 min). This is consistent with our prior anatomical finding that 55% of VTA neurons are TH(+). To directly estimate a false negative rate for our ICC method we recorded VTA neurons from mice in which EGFP production is driven by the TH promoter. All 12 EGFP(+) neurons recorded with a K-gluconate internal solution (as used in our rat recordings) were strongly labeled by TH ICC (recording duration 16.6±1.8 min). However, using recording electrodes with an internal solution with high Cl^−^ concentration reduced the intensity of TH co-labeling, in some cases to background (recording duration 16.7±0.9 min; n = 10). Thus TH is a highly reliable molecular marker for DA neurons in VTA patch clamp recordings provided compatible microelectrode solutions are used.

## Introduction

Powerful aspects of *in vivo* and *ex vivo* electrophysiological techniques include that they can reveal neural activity correlated with specific behaviors, sorting of neural properties by efferent and afferent connections, and sensitivity of particular circuit elements to endogenous and exogenous chemical stimulation. However, the strength of the interpretation of such studies depends on the extent and accuracy of the phenotypic information available for each recorded neuron. That is, the more that is known about the circuit in which the recorded neuron participates and the neurotransmitters and neuromodulators it releases, the more informative electrophysiological observations become.

The certainty with which circuit elements can be identified varies greatly throughout the brain. Neurons in many brain regions, particularly those in subcortical reticular structures, can be highly heterogeneous in neurotransmitter content and connectivity but similar in their electrophysiological properties. This makes it imperative to use molecular markers in conjunction with electrophysiology to fully understand how individual neurons function within defined circuits.

The midbrain VTA is a critical focus of research on neuronal mechanisms of motivation and reinforcement. A major factor for this focus is that the VTA is the single source of DA projections to forebrain regions implicated in these processes. Both *in vivo* and *ex vivo* electrophysiological studies of VTA neurons have been instrumental in forwarding our understanding of motivation and reinforcement, however, approximately 45% of VTA neurons are not DAergic [1]. Tyrosine hydroxylase (TH) is the rate limiting enzyme in the formation of both DA and norepinephrine, and since there are no noradrenergic neurons in the VTA, TH antibodies have emerged as a gold-standard for identifying DA neurons in this brain region. Nonetheless, because of the time, expense and technical challenge of combining ICC with single cell electrophysiology many investigators have sought simple pharmacological or electrophysiological markers that can substitute for post-hoc TH ICC. Several of these markers (inhibition by DA D2 receptor (D2R) activation, long action potential duration, and the presence of a hyperpolarization activated cation current (*I*_h_)) have become accepted as indirect markers and are currently widely used [2], [3], [4]. Unfortunately, many of these markers are present in TH(−) VTA neurons [1], [5], [6], thus calling into question many of the conclusions of research on putative DA neurons. On the other hand, ICC can be unreliable and false negative results are common, thus many investigators continue to use indirect markers under the assumption that the reports that such markers are unreliable arise from technical problems with the TH ICC. In fact, a recent paper presented data indicating that TH detection with ICC becomes severely impaired within minutes of the onset of patch recording in neurons identified as DAergic using independent techniques [7]. In the present paper we identify the technical problem leading to the reported rapid loss of TH ICC in recorded neurons and present evidence that when done with a compatible method, TH staining in recorded neurons is highly reliable. Furthermore, a review of the literature indicates that the other currently used methods for identifying VTA dopamine neurons are associated with a greater likelihood of false negatives than TH ICC. Consequently, until a better method is found, TH ICC should remain the gold standard for identification of VTA DA neurons.

## Results

**Figure 1:**
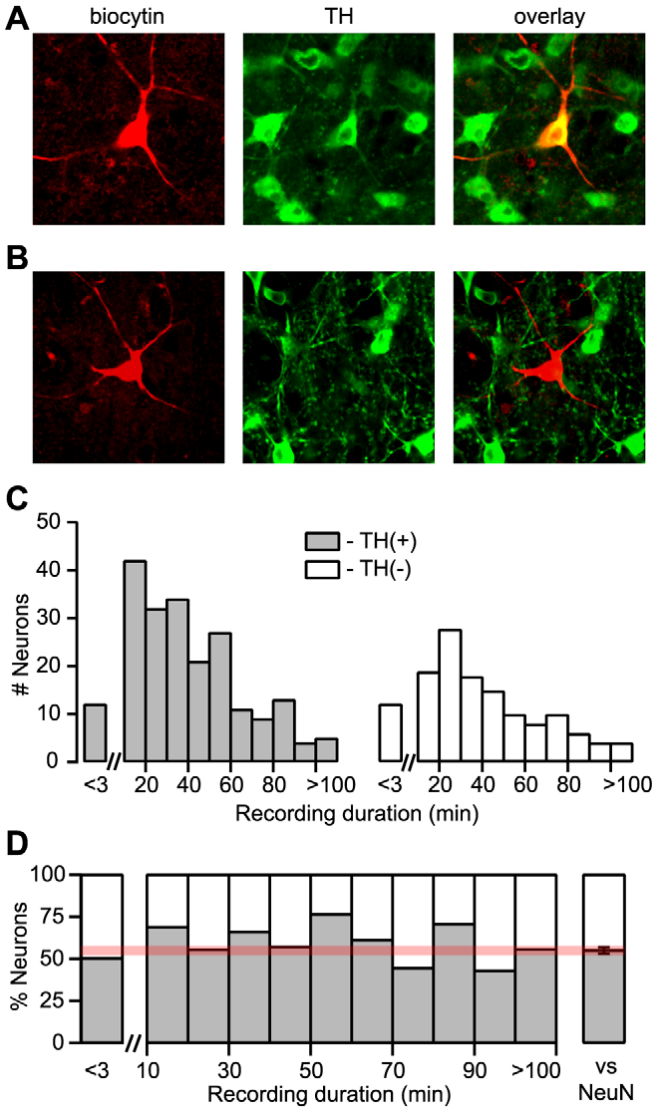
The percent of TH(+) neurons does not decrease with increased recording duration. Examples of rat VTA neurons in which brief whole cell recordings were made (<3 min) and post-hoc immunocytochemical detection revealed that example neuron (A) was TH(+) and example neuron (B) was TH(−). (C) The raw distributions of recording times among neurons determined to be TH(+) or TH(−) with post-hoc TH ICC are similar. (D) The ratio of TH(+) to TH(−) neurons is not related to recording duration, but is very similar to the previously published determination that 55% of all neurons in the VTA (labeled with a NeuN antibody) are TH(+) [1].

We first investigated whether recording in the same configuration that we previously used to compare the properties of DA and non-DA VTA neurons [1], but for a much shorter duration (less than 3 minutes), would result in a higher detection rate of TH in recorded neurons. We chose this 3 minute time point because a recent report suggested that recording from neurons for greater than 15 minutes, but not less than 3, impairs immunocytochemical detection of TH [7], however none of our prior experiments were this brief. As in our previous studies of rat VTA neurons, filled neurons were only analyzed for TH staining if surrounding neurons at the same depth in the tissue exhibited TH ICC signal, ensuring antibody penetration to the level of the filled cell. At less than 3 minutes, we recovered TH(+) (Figure 1A) and TH(−) (Figure 1B) neurons. In fact, exactly half (12/24) of the filled cells were TH(+) (Figure 1C,D). We also sorted all previous experiments with these recording conditions and TH immunocytochemical identification by recording duration to determine if there was a relationship between recording duration and percent TH(+). We hypothesized that longer recording durations would increase the likelihood of a neuron being cytochemically TH(−). However, we detected no evidence of degradation of TH staining over time, even in experiments exceeding 100 minutes (Figure 1C,D). For all recording durations, the % TH(+) was very close to our previous anatomical estimate of 55% of VTA neurons being TH(+) (Figure 1D) [1].

While these data suggested that there is no significant loss of TH staining in rat VTA neurons for the range of recording durations used in our experiments, this result did not directly address the question of whether a significant number of neurons identified as TH(−) are methodological artifacts (false negatives), or true non-DA neurons. It was still possible that TH degrades in even the briefest of recordings, and that this degradation of TH signal reaches a steady state in less than 3 minutes. We therefore completed additional experiments in mice that express EGFP under the TH promoter, enabling us to detect EGFP prior to recording from a neuron. In this way, we established *a priori* that all recorded EGFP(+) neurons should be TH(+). This technique was used recently to suggest that after just 15 minutes of recording, most VTA DA neurons lose their TH immunoreactivity [7]. One obvious difference between this recent report and our methods is the composition of the internal solution: our solution includes an estimated physiological concentration of Cl^−^ (8 mM), while the solution in the previous report included an elevated Cl^−^ concentration (124 mM). We hypothesized that it was this difference in the internal solutions that caused the differences in TH detection, and therefore also attempted to replicate the previous report using the identical KCl internal solution. To ensure that any differences in ICC were not related to slice health or inter-animal variability, for 2 out of the 4 mice used, K-gluconate and KCl-based internal solution use was alternated between slices from the same mouse.

**Figure 2:**
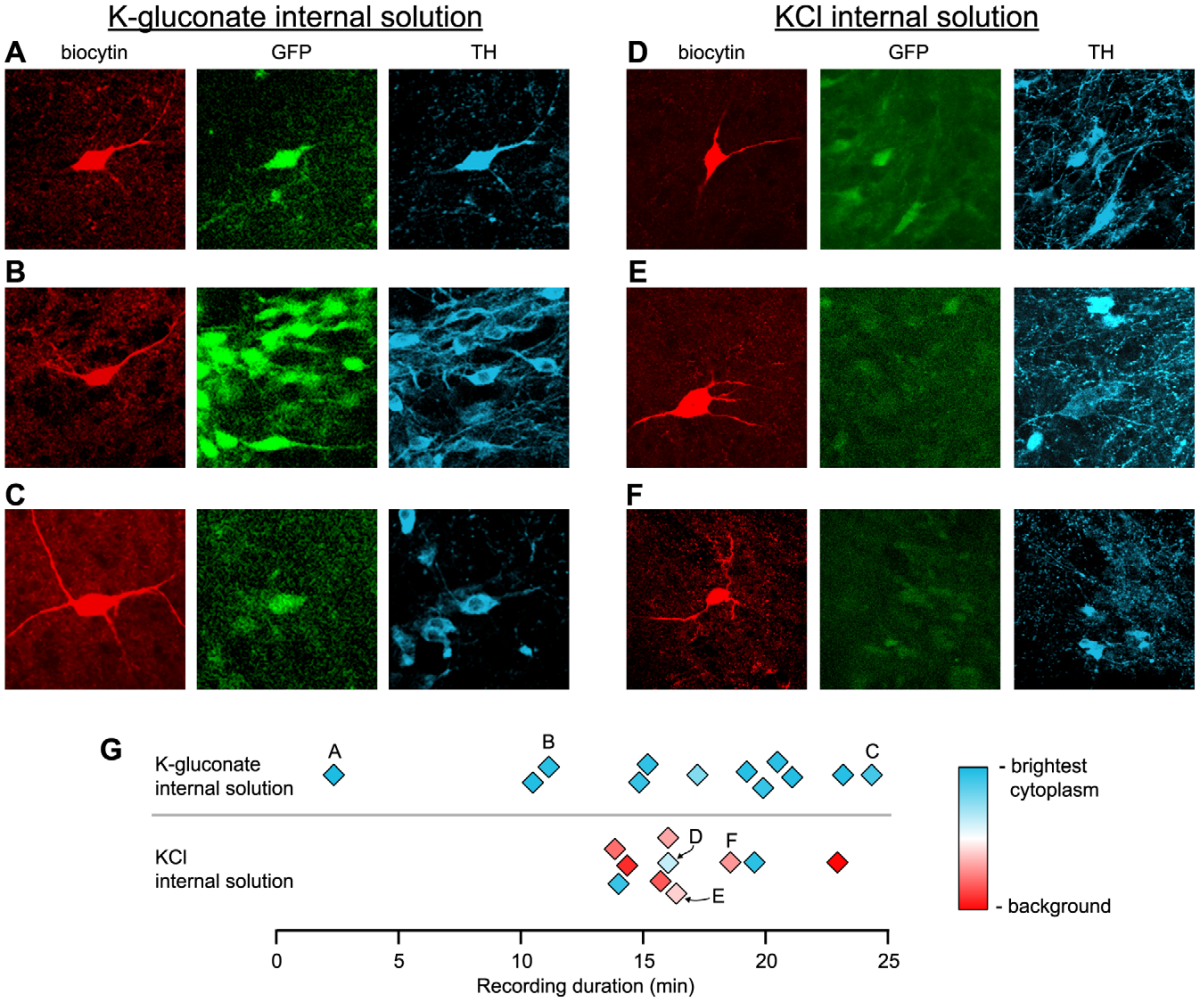
Whole cell recording solution composition influences the preservation of TH in VTA neurons. Recordings were made in VTA EGFP-expressing neurons in tissue from mice where EGFP is expressed under the TH promoter. With the K-gluconate internal solution, TH was strongly detected in all recorded neurons, regardless of recording duration, but not with a KCl internal solution. Examples of K-gluconate filled cells and ICC against EGFP and TH following brief (A), medium (B) and longer (C) duration recordings show clear TH labeling. Examples of KCl filled cells show that the TH signal in these neurons can be discernable (D), very weak (E), or undetectable (F). (G) A within section relative TH ICC intensity was calculated for each filled cell (eq. 1); while neurons recorded with the KCl solution lose TH intensity, neurons recorded with K-gluconate maintain TH intensity.

We recorded from 12 EGFP-labeled VTA neurons with our K-gluconate internal solution as for the rat data described above, and then completed ICC against TH. We quantified the quality of TH ICC signal in the recorded neuron by comparing the TH intensity in the cytoplasm of the recorded neuron to that in the brightest neighboring cell body (eq. 1). Of the 12 EGFP-labeled neurons, all strongly expressed TH immunocytochemical signal, regardless of recording duration (Figure 2A,B,C,G). When we recorded from EGFP(+) VTA neurons using the KCl-based internal solution described by Zhang et al. [7], we found that the TH immunocytochemical signal was drastically degraded, and in some cases not discernable from background labeling (Figure 2D,E,F,G). Interestingly, the slices in which recordings were made with KCl also suffered from EGFP degradation, not only in the recorded neuron but in the surrounding neurons (Figure 2D,E,F), yet in slices from the same mouse in which K-gluconate was used we did not observe this loss of EGFP signal.

## Discussion

Accurate identification of neural phenotypes is critical to understanding how central nervous system circuits function. In the VTA, methods that appear to be sensible surrogates for direct identification of DA neurons are widely employed including long action potential duration, hyperpolarization in response to DA D2R activation, and *I*_h_. In particular, testing D2R responses and *I*_h_ expression are attractive because they provide a clear binary delineation between responsive, putative DA neurons and non-responsive, putative non-DA neurons. We previously reported that significant numbers of TH(−) (putative non-DA) neurons are also inhibited by D2R activation and express an *I*_h_; this result could reflect either that the widely accepted criteria for identifying VTA DA neurons are inaccurate, or that our data includes many neurons misidentified as non-DA. While it would be technically easier to study the organization and physiology of the VTA if the indirect criteria were accurate, especially for *in vivo* recording, accumulating data suggests that these are not reliable markers for VTA DA neurons. For instance, it is clear that a subset of VTA DA neurons express very short duration action potentials [1], [8], [9], violating the tenet that VTA DA neurons have long duration action potentials. Several groups have reported that there are also VTA DA neurons in rats and mice that are not hyperpolarized by D2R activation [1], [8], [9]. Therefore action potential duration and D2R agonist responses clearly do not differentiate DA from non-DA neurons in the VTA; classifying such neurons as non-DAergic via these unreliable criteria will lead to significant errors of omission.

In this work we directly tested the strength of our argument that identifying VTA neurons based on AP duration, D2R agonist responses, or *I*_h_ size produces errors of commission. We found that only when the Cl^−^ concentration within the patch electrode (and therefore likely in the recorded cell) is high does the ability to immunocytochemically detect TH rapidly degrade with whole cell recording. One distinct possibility is that this high Cl^−^ concentration initiates the phosphorylation of TH through one of a variety of mechanisms [10], [11]. Therefore it is not whole cell recording itself, but only selective conditions utilized in some experiments, that cause a degradation of the TH signal. Since 100% of the EGFP(+) neurons that we recorded from with our K-gluconate internal solution displayed strong immunocytochemical staining for TH, so intense in fact that 7 out of 12 recorded cells exhibited the brightest TH signal in the field of its confocal scan, the data reported here strongly argue against TH degradation causing significant numbers of DA neurons to be misidentified as non-DA neurons in our previous work. While it is the case that false negatives can also result from a failure of an antibody to penetrate the tissue to the level of the filled cell, this problem is easily recognized by the absence of TH staining in other neurons in that focal plane. In fact, because not all TH(+) VTA neurons are EGFP(+) in TH-EGFP mice and TH mRNA is negative in many EGFP(+) neurons in these mice, Zhang and colleagues concluded, and we agree, that, when positive, the most reliable current marker for VTA DA neurons is in fact TH ICC [7].

Some of our previously published data also support the robust nature of post-hoc TH ICC. If our experiments suffered from either TH degradation that depended on the duration of the whole cell recordings or a consistent % of false negative data independent of recording duration, those data sets would look quite different. For instance, we reported that kappa opioid receptor (KOR) activation causes hyperpolarization only in TH(+) neurons [12], and that all TH(+) neurons projecting to either the medial prefrontal cortex or the amygdala are inhibited by KOR activation [8], [13]. Across all experiments where we have tested neural responses to KOR agonists and completed ICC for TH, 32/32 KOR-inhibited neurons were TH(+). These 32 neurons were recorded for durations ranging from 20.6 min to 122.7 min, (median = 52.6 min). If TH protein degraded significantly in recordings of longer durations, or if some other technical issue was producing false negative TH detection, then at least some of these neurons should have been TH(−).

**Figure 3:**
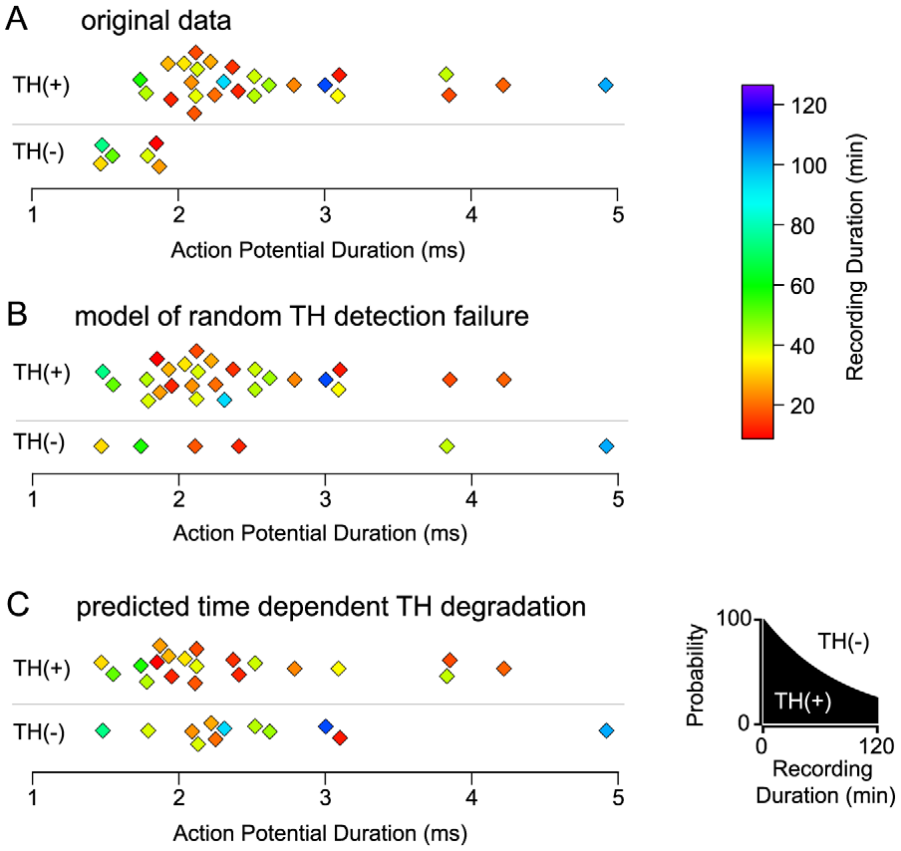
False negative immunocytochemical data do not contaminate our prior data. (A) Among NAc-projecting VTA neurons, we previously demonstrated that TH(+) neurons exhibited longer duration action potentials than TH(−) neurons [6]. Here we show this finding is independent of recording duration. (B) The same physiological data plotted under the premise that TH(−) neurons arise from a random technical failure. We used a time-independent probability of 0.19 for TH(−) neurons consistent with the actual data presented in (A). (C) The same data replotted utilizing a time dependent model of TH degradation (eq. 2). The probability that a neuron was TH(+) or TH(−) was modeled by a single exponential decay, and for each neuron the probability that it would be TH(+) was calculated based on the recording duration (right). A random number was then generated to determine whether a neuron fell on the TH(+) or TH(−) side of the probability plot.

Another data set we have published that indicates our techniques yield a very low false negative rate is the sorting of action potential duration of NAc-projecting VTA neurons by TH content. *In vivo*, Yim and Mogenson demonstrated that among VTA neurons that project to the NAc, those with longer action potential durations also exhibited slower axon conduction velocities, suggesting that these were DA neurons with unmyelinated axons [14]. The neurons with shorter action potential durations had faster conduction velocities, consistent with being non-DA neurons with myelinated axons. We previously reported parallel results using *ex vivo* techniques: we recorded from VTA neurons retrogradely labeled by injections into the NAc and compared the action potential durations of TH(+) and TH(−) neurons. We found not only that action potential durations in TH(+) neurons were significantly longer than in TH(−) neurons, but also that there was almost no overlap in action potential duration between TH(+) and TH(−) NAc-projecting neurons (Figure 3A) [8]. Although the recording durations vary widely, there is no apparent relationship between recording duration and detected TH content (Figure 3A). If we take the same physiology data but assume that all of the neurons were in fact DAergic and that TH(−) cells arise from a technical issue that can affect any VTA neuron with an equal probability, the TH(−) neurons would express action potential durations that are intermixed with the TH(+) neurons (Figure 3B). Similarly, if there was time dependent degradation of TH that caused detection of TH(−) neurons, the action potential durations would again lack any clear sorting in this sample (Figure 3C).

The neurons of the VTA vary in neurotransmitter content, afferent input sources, projection target, pharmacological responses, membrane properties, and firing patterns *in vivo*. While direct immunocytochemical identification of DA neurons is not always technically possible, it is currently the least likely method to cause false negative identification of DA neurons in the VTA, and we demonstrate here that it can be quite robust and durable with the appropriate recording conditions.

## Materials and Methods

All animal protocols were conducted under National Institutes Health (NIH). 

Guidelines using the NIH handbook Animals in Research and were approved by the Institutional Animal Care and Use Committee (Ernest Gallo Clinic and Research Center, University of California at San Francisco, Emeryville, CA), approval ID 10.01.202.

### Slice Preparation and Electrophysiology

Male Sprague Dawley rats (20–40 days old) or 2 month old TH-EGFP mice were anesthetized with isoflurane, decapitated, and the brains were removed. Horizontal brain slices (150 µm thick) containing the VTA were prepared using a vibratome (Leica Instruments). Slices were submerged in Ringer solution containing (in mM): 119 NaCl, 2.5 KCl, 1.3 MgSO_4_, 1.0 NaH_2_PO_4_, 2.5 CaCl_2_, 26.2 NaHCO_3_, and 11 glucose, saturated with 95% O_2_ - 5% CO_2_ and allowed to equilibrate at 35°C for at least 1 hour.

Individual slices were visualized under a Zeiss Axioskop with differential interference contrast optics, infrared illumination, and epiflourescence (HBO 100), utilizing a Zeiss Axiocam MRm and Axiovision 4 software. Whole cell patch clamp recordings in rat brain slices were made at 31°C using 2.5–5 MΩ pipettes containing (in mM): 123 d-gluconic acid, 10 HEPES, 0.2 EGTA, 8 NaCl, 2 MgATP, and 0.3 Na_3_GTP (pH adjusted to 7.2 with 4 M KOH, osmolarity adjusted to 275). Biocytin (0.1%) was added to the internal solution in order to mark the recorded neuron for later cytochemical characterization. In brain slices from TH-EGFP mice, all recorded neurons were identified as EGFP-expressing prior to patching. Neurons were recorded with either the identical solution used for the recordings from rat VTA neurons, or the internal solution described by Zhang et al. [7] (in mM): 120 KCl, 0.2 EGTA, 10 HEPES, 2 MgCl_2_, with the same concentration of biocytin, ATP, and GTP, pH, and osmolarity as the K-gluconate solution.

Recordings were made using an Axopatch 1-D (Molecular Devices), filtered at 2 kHz and collected at 5 kHz using IGOR Pro (Wavemetrics). Immediately following breaking in to the cell, *I*_h_ was measured by voltage clamping cells and stepping from −60 to −40, −50, −70, −80, −90, −100, −110, and −120 mV. Then neurons were held in current clamp (I = 0) for the remainder of the experiment. For recordings in rat neurons with durations less than 3 minutes, the time was determined from the moment of achieving whole cell configuration to the time the recording electrode was withdrawn from the cell. In all other cases, the reported duration is the elapsed time recorded in current clamp following *I*_h_ measurement.

ATP, GTP, biocytin, salts, and other reagents were obtained from Sigma Chemical. The TH-EGFP mice (strain Tg(Th-EGFP)21-31Koba) utilized here are available through RIKEN BRC.

### Immunocytochemistry

Following recordings, slices containing biocytin fills were fixed in 4% paraformaldyhyde for 2 hrs, and then washed 5 times with PBS (pH 7.4). Slices were pre-blocked in PBS containing 0.3% (v/v) Tween20, 0.2% BSA, and 5% normal goat serum for 2 hours at room temperature. Samples were then agitated for 48 hours at 4°C with rabbit anti-tyrosine hydroxylase polyclonal antibody (1∶100) and chicken anti-GFP antibody polyclonal (1∶2000). The slices were then washed thoroughly in PBS with 0.3% Tween20 and 0.2% (w/v) BSA before being agitated overnight at 4°C with Cy5 anti-rabbit secondary antibody (1∶100), FITC anti-chicken antibody (1∶100) and Hilyte Fluor 555 labeled streptavidin (6.5 uL per 1 mL) (AnaSpec Inc.). Sections were mounted on slides using VectaShield anti-fade mounting media (Vector Inc.) and visualized under a Zeiss LSM510 META microscope. In all sections it was required that TH antibody labeled neighboring neurons at the same depth in the slice as the filled cell in order to evaluate the fill as TH(+) or TH(−).

Primary antibodies were obtained from Chemicon International, secondary antibodies from Jackson ImmunoResearch Laboratories, and all other reagents from Sigma Chemical.

### Data Analysis

In the experiments utilizing TH-EGFP mice, we calculated the relative brightness of TH ICC in biocytin-labeled neurons by analyzing fluorescent signal intensity using the image analysis software ImageJ (NIH). The normalized intensity of fluorescent signal in the biocytin-filled cell body (*NN_i_*) was calculated as 
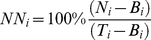
(1)


where *N_i_* was the raw intensity of the TH fluorescence in the filled neuron, *T_i_* was the intensity of TH ICC in the brightest TH(+), biocytin-free neuron in the confocal scan, and the background signal intensity in an area of comparable size to the recorded neuron's soma (*B_i_*) was subtracted from each; *i* is the index of the neuron. In 7 cases among the K-gluconate internal solution recordings, *NN_i_* >100% because the recorded neuron was the brightest neuron in the field. In these cases the intensity of TH staining was set to 100%.

For the analysis of TH outcomes predicted by time dependent TH degradation, we started with the dataset of action potential durations in nucleus accumbens (NAc)-projecting VTA neurons for which we had TH immunocytochemical data. Because the TH degradation we observed in the filled cells from the TH-EGFP mice suggested variability in the rate of TH decay, we used a probability function to generate a model of what a time dependent TH degradation would look like in this dataset. We generated a probability plot based on the prediction that 100% of neurons would be TH(+) when the recording duration was 0 minutes, 50% of the neurons would be TH(+) at 60 minutes, and constrained the decay to approach 0. We selected these model parameters in order to yield a reasonable number of predicted TH(−) neurons compared to the actual ICC results. These points were fit with a single exponential curve, as would be expected for a basic diffusion or chemical decay mechanism, yielding the equation
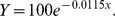
(2)


We then generated random numbers (0–100) for each neuron, and if the random number fell within the TH(+) range for the experiment duration on the probability plot, the neuron was coded TH(+).

For the analysis of the same data using a constant probability of TH co-labeling, the probability 0.19 was used because it is equivalent to the 6/32 neurons in the original dataset that were TH(−). A separate set of random numbers were generated for this analysis (0–1), and random numbers exceeding 0.19 coded neurons as TH(+) and numbers below 0.19 coded neurons as TH(−).

## References

[1] Margolis EB, Lock H, Hjelmstad GO, Fields HL (2006). The ventral tegmental area revisited: is there an electrophysiological marker for dopaminergic neurons?. J Physiol.

[2] Ahn KC, Bernier BE, Harnett MT, Morikawa H (2010). IP3 receptor sensitization during in vivo amphetamine experience enhances NMDA receptor plasticity in dopamine neurons of the ventral tegmental area.. J Neurosci.

[3] Roesch MR, Calu DJ, Schoenbaum G (2007). Dopamine neurons encode the better option in rats deciding between differently delayed or sized rewards.. Nat Neurosci.

[4] Iniguez SD, Vialou V, Warren BL, Cao JL, Alcantara LF (2010). Extracellular signal-regulated kinase-2 within the ventral tegmental area regulates responses to stress.. J Neurosci.

[5] Luo AH, Georges FE, Aston-Jones GS (2008). Novel neurons in ventral tegmental area fire selectively during the active phase of the diurnal cycle.. Eur J Neurosci.

[6] Cameron DL, Wessendorf MW, Williams JT (1997). A subset of ventral tegmental area neurons is inhibited by dopamine, 5-hydroxytryptamine and opioids.. Neuroscience.

[7] Zhang TA, Placzek AN, Dani JA (2010). In vitro identification and electrophysiological characterization of dopamine neurons in the ventral tegmental area.. Neuropharmacology.

[8] Margolis EB, Mitchell JM, Ishikawa J, Hjelmstad GO, Fields HL (2008). Midbrain dopamine neurons: projection target determines action potential duration and dopamine D(2) receptor inhibition.. J Neurosci.

[9] Lammel S, Hetzel A, Hackel O, Jones I, Liss B (2008). Unique Properties of Mesoprefrontal Neurons within a Dual Mesocorticolimbic Dopamine System.. Neuron.

[10] Wertheimer EV, Salicioni AM, Liu W, Trevino CL, Chavez J (2008). Chloride Is essential for capacitation and for the capacitation-associated increase in tyrosine phosphorylation.. J Biol Chem.

[11] Lindgren N, Goiny M, Herrera-Marschitz M, Haycock JW, Hokfelt T (2002). Activation of extracellular signal-regulated kinases 1 and 2 by depolarization stimulates tyrosine hydroxylase phosphorylation and dopamine synthesis in rat brain.. Eur J Neurosci.

[12] Margolis EB, Hjelmstad GO, Bonci A, Fields HL (2003). Kappa-opioid agonists directly inhibit midbrain dopaminergic neurons.. J Neurosci.

[13] Margolis EB, Lock H, Chefer VI, Shippenberg TS, Hjelmstad GO (2006). Kappa opioids selectively control dopaminergic neurons projecting to the prefrontal cortex.. Proc Natl Acad Sci U S A.

[14] Yim CY, Mogenson GJ (1980). Electrophysiological studies of neurons in the ventral tegmental area of Tsai.. Brain Res.

